# Salvianolic Acid B Strikes Back: New Evidence in the Modulation of Expression and Activity of Matrix Metalloproteinase 9 in MDA-MB-231 Human Breast Cancer Cells

**DOI:** 10.3390/molecules27238514

**Published:** 2022-12-03

**Authors:** Andrea Ianni, Pierdomenico Ruggeri, Pierangelo Bellio, Francesco Martino, Giuseppe Celenza, Giuseppe Martino, Nicola Franceschini

**Affiliations:** 1 Department of BioScience and Technology for Food, Agriculture and Environment, University of Teramo, Via Renato Balzarini 1, 64100 Teramo, Italy; 2 Department of Biotechnological and Applied Clinical Sciences, University of L’Aquila, Via Vetoio 1, 67100 L’Aquila, Italy; 3 Department of Cardiovascular, Respiratory, Nephrological, Anaesthetic and Geriatric Sciences, “La Sapienza” University of Rome, Policlinico Umberto I, 00185 Rome, Italy

**Keywords:** Salvianolic acid B, matrix metalloproteinase 9, gelatinase B, MDA-MB-231 human breast cancer cells, TIMP-1

## Abstract

Salvianolic acid B (SalB) is a bioactive compound from *Salviae miltiorrhizae*, one of the most important traditional herbal medicines widely used in several countries for the treatment of cardiovascular diseases. The aim of this study was to evaluate the in vitro effect of SalB on the expression and the activity of matrix metalloproteinase 9 (MMP-9), a zinc-dependent proteolytic enzyme, in human MDA-MB-231 breast cancer cells. This cellular model is characterized by a marked invasive phenotype, supported by a high constitutive expression of MMPs, especially gelatinases. SalB was first of all evaluated by in silico approaches primarily aimed at predicting the main pharmacokinetic parameters. The most favorable interaction between the natural compound and MMP-9 was instead tested by molecular docking analysis that was subsequently verified by an enzymatic inhibition assay. MDA-MB-231 cells were treated with SalB 5 µM and 50 µM for 24 h and 48 h. The conditioned media obtained from treated cells were then analyzed by gelatin zymography and reverse zymography to, respectively, evaluate the MMP-9 activity and the presence of TIMP-1. The expression of the enzyme was then evaluated by Western blot on conditioned media and by analysis of transcripts through reverse transcriptase-polymerase chain reaction (RT-PCR). The in silico approach showed the ability of SalB to interact with the catalytic zinc ion of the enzyme, with a plausible competitive mode of action. The analysis of conditioned culture media showed a reduction in MMP-9 activity and the concomitant decrease in the enzyme concentration, partially confirmed by analysis of transcripts. SalB showed the ability to modulate the function of MMP-9 in MDA-MB-231 cells. To our knowledge, this is the first time in which the role of SalB on MMP-9 in a highly invasive cellular model is investigated. The obtained results impose further and more specific evaluations in order to obtain a better understanding of the biochemical mechanisms that regulate the interaction between this natural compound and the MMP-9.

## 1. Introduction

The MMP-9/Gelatinase B belongs to the wide family of zinc dependent endopeptidases named matrix metalloproteinases (MMPs). MMPs are involved in the physiological degradation of the extracellular matrix (ECM), a fundamental process for tissue development, morphogenesis, remodeling and repair [1]. Due to their important physiological role, MMPs are tightly regulated: their expression is transcriptionally influenced by growth factors, hormones and cytokines, while their activity is tuned by the activation of propeptides (zymogens) and by inhibition of the enzymatic activity mediated by endogenous tissue inhibitors (TIMPs) [2].

Disturbances of this complex network hindering the normal MMPs function are known to be associated with the development of pathological events related to excessive or insufficient ECM turnover: arthritis, wound healing disorders, fibrotic diseases and cancer [3]. 

Regarding cancer, it is known that the proteolytic activity which contributes to the degradation of ECM and basal membrane represents a key step in cell invasion, metastasis and angiogenesis [4]. Moreover, several studies have posed evidence of the crucial role of gelatinases in numerous invasive cancers, such as breast cancer [5,6]. Recent observations also provide evidence that MMPs modulate various aspects of inflammation, some of which seem to be essential for the suppression of innate immune response against tumor cells; in this context, MMPs may exert an immune regulatory function in tumor microenvironment, helping cancer cells to escape immune surveillance [7]. It is easy to understand that the identification of MMP inhibitors (MMPi) today represents an important opportunity for the treatment and prevention of numerous chronic and life-threatening diseases. For these reasons, MMPs inhibitor drug discovery has emerged as an important area of research in many fields of medical sciences as also attested by the discovery or repositioning of several MMPs inhibitors [8,9,10].

In the last decade, great interest was generated in the characterization and use of natural compounds credited with high bioactive potential, including anti-inflammatory, antibacterial, antioxidant, anti-cancer and anti-diabetic properties [11,12,13]. Into this scenario fits the interest in the identification of natural compounds capable of controlling the expression and activity of MMPs. Specifically, flavonoids have been found to influence MMPs levels in different ways. In many cell types, flavonoids have been described to down-regulate MMPs biosynthesis. Quercetin, for instance, was reported to inhibit the invasivity of murine melanoma cells by decreasing pro-MMP-9 via PKC pathway [14]. It is also known that certain MMPs can be activated by oxidative stress and the antioxidative effect of flavonoids may influence this mechanism [15]. 

*Salvia miltiorrhiza*, also known as red sage or Chinese sage, is one of the most widely used traditional herb medicines recommended for the treatment of a variety of diseases, such as cardiovascular diseases, hepatitis, hepatocirrhosis, chronic renal failure and dysmenorrhea. In *S. miltiorrhiza,* two pharmacologically active fractions were identified: lipophilic diterpenoids transhinones and water-soluble phenolic acids [16]. In recent years, research has been focused on the phenolic acid fraction where twenty-five phenolic acid compounds have been isolated and identified. Specifically, Salvianolic acid B (SalB) represents the most abundant compound, accounting for 3–5% of total dried weight [17]. To date, SalB has been identified as an agent which may be useful in controlling the expression of MMP-2 and MMP-9 in pathological conditions involving the cardiovascular system. In this context, we should cite the inhibition of MMP-2 upregulation in human aortic smooth muscle cells via suppression of NAD(P)H oxidase-derived reactive oxygen species [18], the prevention of the infarct-induced cardiac remodeling through competitive inhibition of MMP-9 [19] and the attenuation of cardiac fibroblast migration, collagen and cytokine secretion through the in vitro inhibition of the catalytic domain of MMP-9 [20]. However, little attention has been paid to the role of SalB in tumor cell lines characterized by high invasivity potential, in which MMPs play a leading role. The aim of this work is therefore to verify the effect of SalB in regulating the in vitro function of MMP-9 in MDA-MB-231 human breast cancer cells. The only studies that involved the treatment of MDA-MB-231 cells with SalB focused attention on different biochemical and molecular aspects. For example, the work published by Sha et al. [21] analyzed the effects induced by the natural compound on the cell viability, cell cycle and apoptosis of triple-negative MDA-MB-231 cells with the hormone receptor-positive MCF-7 cells as the control. The main finding of the work was specifically related to SalB ability to enhance the cell apoptosis and decrease cell proliferation by regulating the ceramide glycosylation enzymes. Overall, most of the studies performed on human breast cancer cells involved the use of non-invasive phenotypes (MCF-7 cells), confirming the role of SalB in cell proliferation, migration and invasion abilities, without specific assessments on the gelatinases function [22].

## 2. Results

### 2.1. In Silico Evaluations and In Vitro Analysis of the Interaction between Salvianolic Acid B and MMP-9

**Figure 1:**
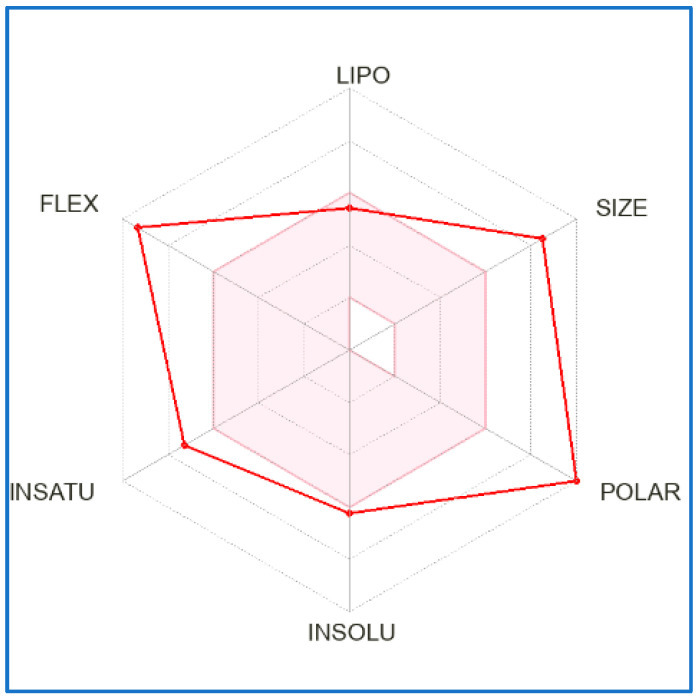
Bioavailability radar chart of SalB. The bioavailability radar gives a first glance on the drug-likeness of the compound, considering a total of six physicochemical properties: lipophilicity, size, polarity, solubility, flexibility and saturation. The pink zone represents the physicochemical space for oral bioavailability, and the red line represents the specific oral bioavailability properties associated with the analyzed compound.

The SwissADME (absorption, distribution, metabolism and excretion) web tool was exploited with the aim to obtain a prediction concerning the pharmacokinetic properties of SalB. The main information of the analysis is reported in Table 1, and indicate low absorption from the gastrointestinal (GI) tract and the inability to permeate the blood–brain barrier (BBB). Furthermore, SalB seems unable to inhibit the activity of cytochrome P450 isoforms (CYP1A2, CYP2C19, CYP2C9, CYP2D6, CYP3A4), whose function is associated with drug elimination through metabolic biotransformation. In Figure 1, the bioavailability radar that gives a first glance on the drug-likeness of the compound is instead reported, considering a total of six physicochemical properties: lipophilicity, size, polarity, solubility, flexibility and saturation. From this point of view, SalB showed itself to be a good candidate with reference to lipophilicity and solubility, while the report highlighted values outside the physicochemical range in the case of size, polarity, flexibility and saturation.

**Figure 2:**
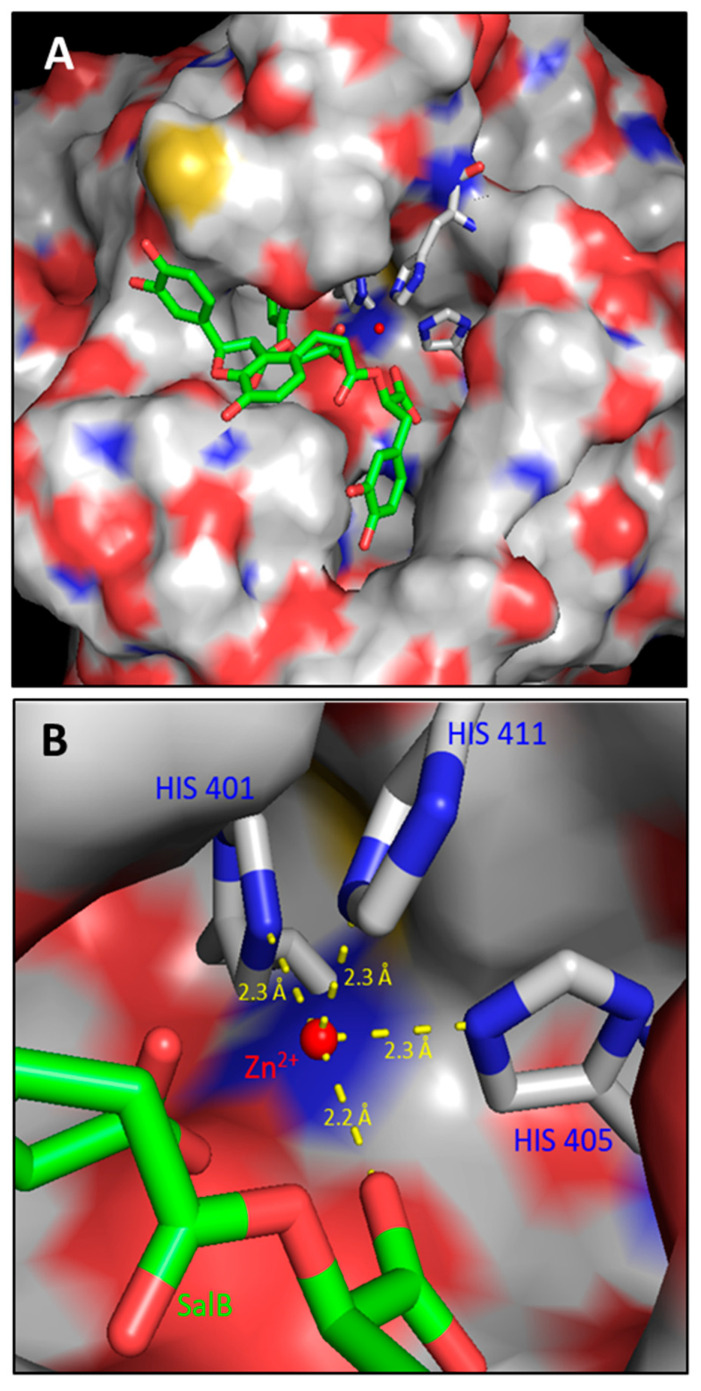
Molecular Docking analysis of the interaction between SalB and MMP-9. (**A**) SalB ability to approach the MMP-9 catalytic pocket. (**B**) Molecular docking of SalB in the catalytic domain of MMP-9 (PDB code: 1GKC). SalB seems able to interact with the catalytic Zn^2+^ ion which is coordinated by three histidines (HIS401, HIS405, HIS411). The analysis specifically suggests the existence of a coordinated bond between the catalytic Zn^2+^ atom and a carboxyl oxygen of SalB structure.

In order to predict the most probable binding conformations between SalB and MMP-9, a preliminary in silico study was performed. The most favorable docking is associated with a ΔG value equal to −14.786 kcal/mol; in this condition (Figure 2A), SalB is able to approach the active site of the enzyme, placing its carboxylic group close to the catalytic zinc ion. In addition to this, the distance between the O6 SalB carboxylic oxygen and the catalytic zinc ion is 2.2 Å (Figure 2B), suggesting the plausible existence of a non-covalent interaction.

**Figure 3:**
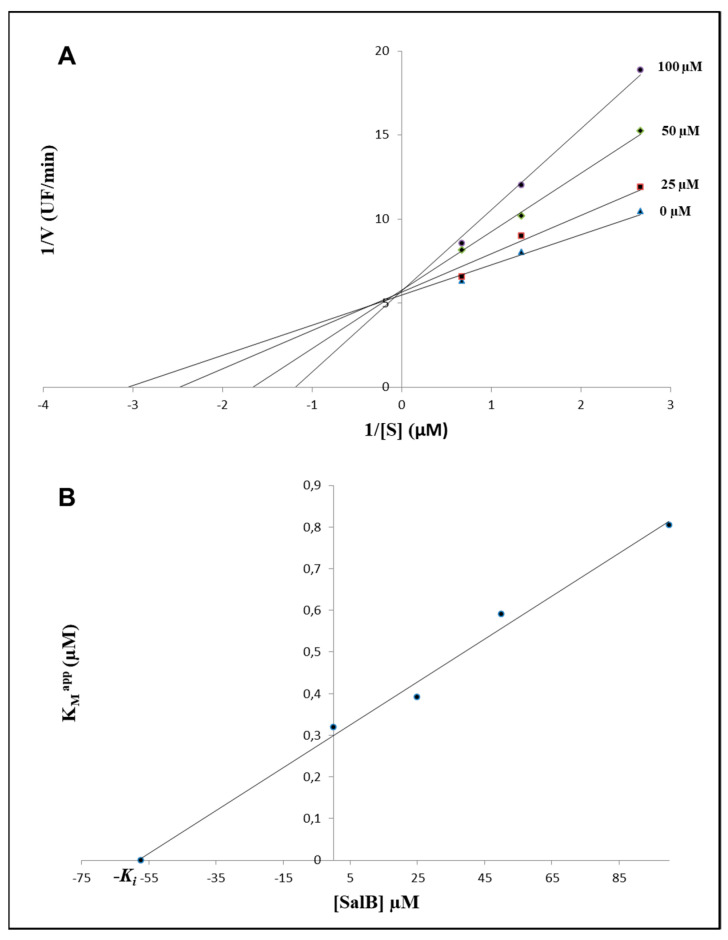
Kinetic analysis of SalB against the catalytic domain of MMP-9. (**A**) Double reciprocal plots of 1/V versus 1/[S] suggest the competitive inhibition of the activity performed by the catalytic domain of MMP-9 (CDMMP-9). (**B**) Secondary plot of K_M_^app^ versus different SalB concentrations. The K_i_ value of SalB against CDMMP-9 was calculated to be equal to 57.37 μM.

To confirm the computational results, an enzymatic inhibition assay was performed. For this purpose, a recombinant active human MMP-9, composed of the catalytic domain, the gelatin binding domain and the metal binding domain (amino acids 107–457), was used. Increasing the concentrations of SalB ranging from 0 μM to 100 μM, in the presence of different concentrations of substrate (0.375–1.5 µM), suggested a competitive inhibition of the activity performed by the MMP-9 catalytic domain (CDMMP-9) (Figure 3A), and allowed to define a K_i_ value equal to 57.37 ± 3.96 µM (Figure 3B).

### 2.2. Evaluation of Gelatinolytic Activity in MDA-MB-231 Cells

The zymographic analysis was performed on conditioned media obtained from MDA-MB-231 cells treated for 24 h and 48 h with SalB 5 µM (SalB-5) and 50 µM (SalB-50). Such analysis was effective in highlighting the gelatinolytic potential attributable to MMP-2 and MMP-9.

**Figure 4:**
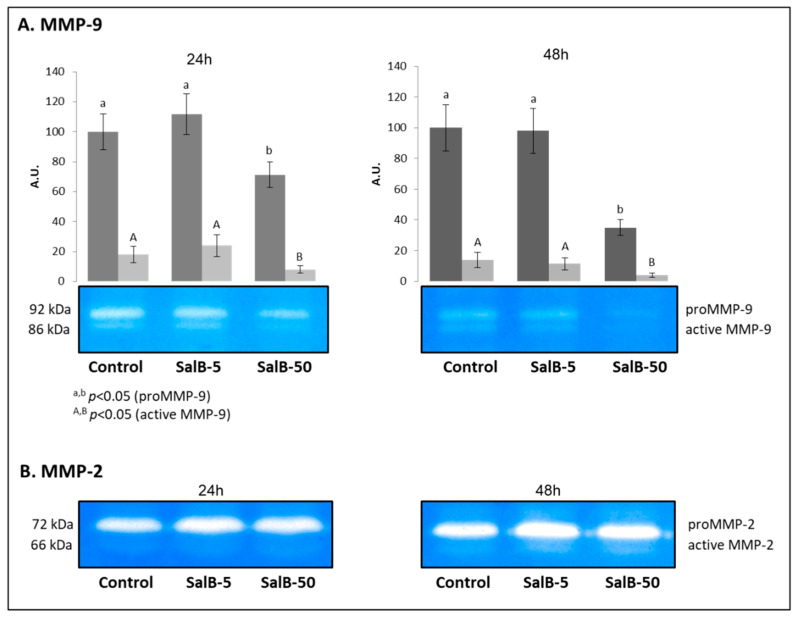
Gelatin Zymography for the evaluation of MMP-9 and MMP-2 activity. Zymographic evaluation of MMP-9 (**A**) and MMP-2 (**B**) activity in conditioned media obtained from MDA-MB-231 cells treated for 24 h and 48 h with SalB 5 and 50 µM.

As shown in Figure 4A, SalB was able to influence the activity of MMP-9, which was significantly reduced in the presence of the higher concentration of the natural compound (50 μM), both after 24 h and 48 h of treatment (*p* < 0.05). The analysis also highlighted the activity of the active form of the enzyme, which follows the same pattern observed for the zymogen, with lower ability to hydrolyze gelatin in SalB-50 samples at both treatment times (*p* < 0.05).

With regard to MMP-2 (Figure 4B), no significant variations were evidenced, both in relation to the dosage and the timing of the treatment (*p* > 0.05).

### 2.3. Salvianolic Acid B Had no Effects on TIMP-1 Expression in MDA-MB-231 Cells

**Figure 5:**
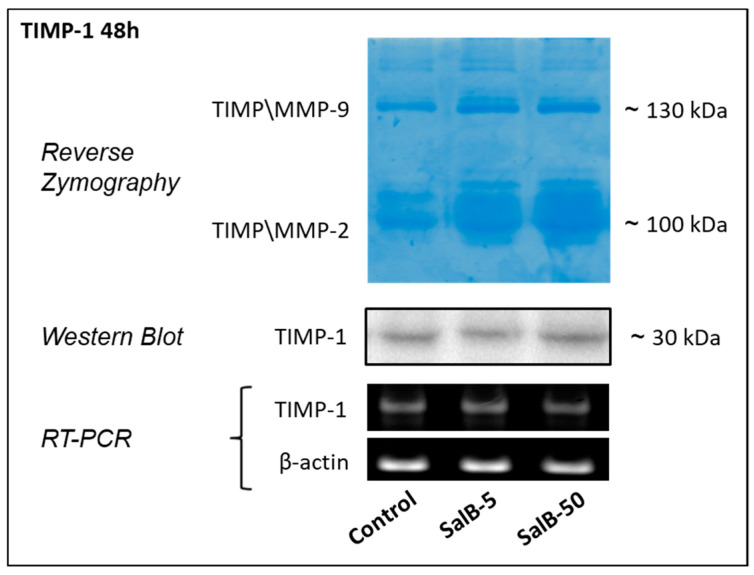
Analysis of TIMP-1 through reverse zymography, Western blot and RT-PCR. The role of the tissue inhibitor of metalloproteinases-1 (TIMP-1) has been evaluated through reverse zymography and Western blot that were performed on conditioned media obtained from MDA-MB-231 cells treated for 48 h with SalB 5 and 50 µM. In the same sampling were also collected cells for transcripts evaluation by RT-PCR.

The potential role of SalB in influencing the endogenous regulation of MMP-9 activity was evaluated by focusing the attention on TIMP-1. Figure 5 shows the results obtained from reverse zymography and Western blot performed on conditioned culture media, and RT-PCR applied on mRNA purified from the corresponding cells. By combining these different approaches, no significant changes (*p* > 0.05) were found in the expression and in the release of TIMP-1 in the extracellular environment.

### 2.4. MMP-9 Expression Was Affected in Treated MDA-MB-231 Cells

**Figure 6:**
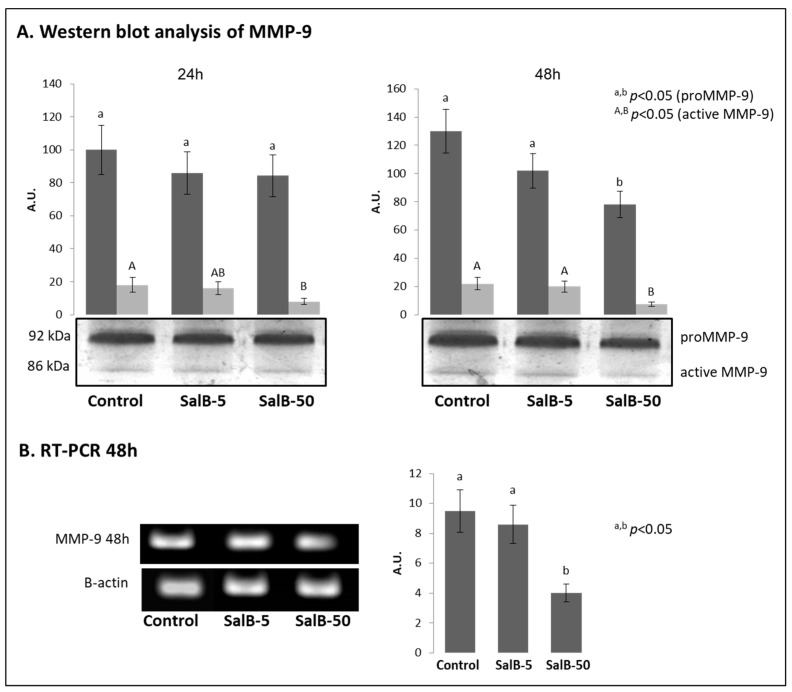
Analysis of TIMP-1 through reverse zymography, Western blot and RT-PCR. The Western blot analysis and RT-PCR of MMP-9. (**A**) The MMP-9 expression was firstly evaluated through Western blot on conditioned media obtained from MDA-MB-231 cells treated for 24 h and 48 h with SalB 5 and 50 µM. Different lower case letters (a,b) indicate significant differences (*p* < 0.05) for the proMMP-9, while different upper case letters indicate significant differences (*p* < 0.05) for the active form of MMP-9. (**B**) The collection of MDA-MB-231 cells was useful to perform the analysis on MMP-9 transcripts after 48 h treatment with SalB 5 and 50 µM.

The samples previously used for the zymographic evaluations, were also subjected to Western blot analysis to verify the relative amount of the enzyme in the conditioned culture media obtained from the various treatments (Figure 6A). In this case, SalB significantly affected the amount of MMP-9 in the conditioned culture medium (*p* < 0.01) only when it was administered at the highest concentration (50 μM) during 48 h treatment. 

In order to verify any changes at the molecular level, the MMP-9 transcripts in cells subjected to 48 h treatment were purified and analyzed. As shown in Figure 6B, a significant reduction in MMP-9 transcript expression (*p* < 0.05) was effectively noted following the treatment with 50 µM SalB, while no variations were evidenced in SalB-5 samples (*p* > 0.05).

## 3. Discussion

Several studies have been conducted to evaluate the ability of SalB to regulate the activity of gelatinases, particularly of MMP-9. To our knowledge this is the first study in which this aspect is evaluated on human MDA-MB-321 breast cancer cells, a highly invasive cellular model characterized by a marked constitutive expression of MMP-9. 

Before the evaluation of SalB effect on the selected cell line, a preliminary in silico study was performed for the prediction of the drug-likeness of the natural compound and the most probable binding conformations between SalB and MMP-9. The SwissADME web tool represents an informative approach in order to obtain a prediction about the biotransformation of specific compounds into drugs. First of all, this evaluation evidenced for SalB low absorption from the gastrointestinal tract and the inability to permeate the blood–brain barrier (BBB). In particular, this last aspect argues in favor of the fact that SalB should not induce adverse effects at the level of the central nervous system. Besides this, SalB does not represent a substrate of the permeability glycoprotein (P-gp) that was suggested to be one of the most relevant members among the ATP-binding transporters, responsible for limiting the oral bioavailability of drugs that act as its substrates [23]. In the sphere of pharmacokinetic parameters, the finding concerning the interaction of the natural compound with cytochromes P450 is also relevant, which is actively involved in the processes of drug elimination through metabolic biotransformation [24]. SalB does not represent an inhibitor of these factors, an aspect of considerable importance considering that the inhibition of these isoenzymes was reported to be one of the main causes of pharmacokinetics-related drug–drug interactions, leading to toxic or other unwanted adverse effects due to the lower clearance and accumulation of the drug or its metabolites [25]. The bioavailability radar generated by the web tool, that gives a first glance on the drug-likeness of the compound, showed overall good attitudes for SalB with reference to lipophilicity and solubility, while conditions outside the physicochemical range were evidenced in the case of size, polarity, flexibility and saturation. This finding testifies to the fact that in the pharmaceutical field it could be more plausible for the use of specific structural domains of the natural compound, rather than the molecule in its entirety.

With reference to the specific interaction between SalB and MMP-9, molecular docking evaluations were performed. Specifically, the most favorable docking is associated with a ΔG value equal to −14.786 kcal/mol; in this condition SalB is able to approach the active site of the enzyme, placing its carboxylic group close to the catalytic zinc ion. In this conformation, a distance of 2.2 Å was calculated between the SalB carboxylic oxygen and the catalytic zinc atom, suggesting the possible onset of a non-covalent interaction between SalB and MMP-9, in addition to the three coordination interactions between zinc and the catalytic histidines (HIS401, HIS405 and HIS411). Such geometrical constraints are therefore compatible with a tetracoordinated chelation model of the catalytic zinc ion as also suggested by Jacobsen et al. [9] who inserted the carboxylic residue in the list of the “zinc binding domains”.

In order to confirm the computational results, an enzymatic inhibition assay was performed by using a recombinant active human MMP-9. The calculated K_i_ value was equal to 57.37 ± 3.96 µM, a finding in full agreement with what has been previously reported by Jiang et al. [19], who found a K_i_ value of 79.2 µM, by using a similar analytical strategy.

The encouraging data obtained by the in silico analysis confirmed by the enzymatic inhibition assay, provided a rationale for the in vitro investigation on human MDA-MB-231 breast cancer cells, characterized by a marked invasive phenotype, supported by a high constitutive expression of several MMPs, especially MMP-9 [26]. 

In order to define the best experimental conditions for cell treatment with SalB, an MTT cell viability assay was performed, allowing us to define the range of concentrations of SalB and the time of exposure in which the cytotoxic effect does not exceed 10% of cell mortality with respect to untreated cells. In our intention, this was performed to make sure that the treated cells maintain the same vitality as the control cells, in order to ensure that any variations in the expression of MMP-9 were to be totally attributed to the effect of SalB and not simply to a reduction in cell viability. Through this preliminary approach, we believe we have given greater strength to the considerations made on the specific effect of the natural compound on the MMP-9 function. Cells were then treated with two concentrations of SalB, 5 µM (SalB-5) and 50 µM (SalB-50), for 24 h and 48 h, respectively.

The zymographic analysis of the conditioned media showed the ability of SalB to inhibit the activity of MMP-9. The analysis also highlighted the activity of the active form of the enzyme, which follows the same pattern observed for the zymogen. This observation suggests that the relation between the two forms remains unchanged under all the experimental conditions and testifies to the fact that SalB had no effect on the pro-enzyme activation mechanism. The observed reduction in activity may therefore depend on a competitive inhibition model based on the prevalent interaction of SalB with the catalytic domain of the enzyme. This hypothesis finds support in the results obtained from the in silico investigation and is in agreement with what was previously observed by Jiang et al. [19] who hypothesized a competitive inhibition of MMP-9 by the SalB. In the same zymograms, the activity of MMP-2 was also visualized. In this case, the SalB did not induce significant variations; a behavior already highlighted in a previous study in which no significant regulation of SalB on MMP-2 activity was found, suggesting that the natural compound was more specific on MMP-9 than on MMP-2 [19]. However, it must be reported that in other studies conducted on murine models or alternative cell lines, a drastic reduction in MMP-2 activity was reported as a consequence of the ability of SalB to interact with mediators of gelatinase expression. One of the best characterized mechanisms concern the inhibition of the tumor necrosis factor-α (TNF-α)-induced MMP-2 upregulation in human aortic smooth muscle cells via suppression of the NAD(P)H oxidase-derived reactive oxygen species [18,27].

It is known that the MMP activity in the extracellular environment is finely regulated by specific endogenous inhibitors (TIMPs) which interact with the enzyme in a stoichiometric 1:1 ratio. For this reason, in the next phase of the study, the role of TIMP-1, which possesses a prevailing selectivity for gelatinases, was evaluated in order to better understand the effect of SalB treatment on MDA-MB-231 cells, and in particular on MMP-9 function. By combining different approaches, no significant changes were found in the expression and in the release of TIMP-1 in the extracellular environment, a fact that confers additional strength to the SalB ability to act as an MMP-9 inhibitor. Concerning the relationship between SalB and TIMPs, no specific studies have been conducted. However, the study performed by Dai et al. [28] must be taken into consideration. They investigated the antifibrotic activity of the active compounds of *Salviae miltiorrhizae* on mice oral mucosal fibroblasts and reported the ability of tanshinone IIA, salvianolic acid A and SalB to reduce TIMP-1 and TIMP-2 expression, a phenomenon that predisposes an increase in gelatinases activity. In agreement with what has been described, it should be also reported that other studies highlighted that flavonoids generally have the ability to influence the expression of TIMPs, with even quite marked effects on the ECM remodeling processes. These are evidence observed in cell lines and in vivo models in which, contextually to the effect on TIMPs, perturbations were also monitored for the expression and activity of other factors involved in crucial signaling pathways such as focal adhesion kinase (FAK), phosphatidylinositol-3-kinase (PI3K)-Akt, signal transducer and activator of transcription 3 (STAT3), nuclear factor κB (NFκB), and mitogen-activated protein kinase (MAPK) [29].

The same samples used for the zymographic evaluations were also subjected to Western blot analysis to verify the amount of the enzyme in the conditioned culture media obtained from the various treatments. In this case, SalB seems to be able to affect the amount of MMP-9 in the conditioned culture medium only if administered at the highest concentration for relatively longer time intervals. This may be due both to a direct effect of SalB in the reduction of gene expression, but also to the ability of the natural compound to interfere with the release of the zymogen from the cytosol to the extracellular environment. For this reason, the MMP-9 transcripts from cells subjected to 48 h treatment with SalB were purified and analyzed in order to highlight any changes in their expression. A significant reduction in MMP-9 expression was effectively noted following the treatment with 50 µM SalB. This therefore confirmed SalB tendency to reach the cytoplasm and interfere with the biochemical mechanisms responsible for regulating the gene expression. In this regard, Lin et al. [27] discussed the ability of SalB to inhibit MMP-2 and MMP-9 expression in LPS-stimulated human aortic smooth muscle cells (HASMCs) advancing the hypothesis of a mechanism based on the suppression of JNK and ERK phosphorylation. This is the first time, in our knowledge, that this aspect is highlighted in invasive tumor cells. Therefore, the specific biochemical mechanisms involved require further and more specific evaluations.

## 4. Materials and Methods

### 4.1. Reagents

The Salvianolic acid B (SalB), dimethyl sulfoxide (DMSO), MTT [3-(4,5-dimethylthiazol-2-yl)-2,5-diphenyltetrazolium bromide], trypan blue, Triton X-100, Tween 20 and type B gelatin were purchased from Sigma-Aldrich Chemical Co. (Milan, Italy). Dulbecco’s modified Eagle’s medium (DMEM), foetal bovine serum (FBS), penicillin, streptomycin, glutamine and trypsin were purchased from Euroclone S.p.A. (Milan, Italy). Human recombinant catalytic domain of MMP-9 was obtained from Vinci Biochem S.r.l. (Firenze, Italy). All other chemicals were reagent grade. Stock solution of SalB (50 mM) was prepared in DMSO and stored in the dark at −20 °C. 

### 4.2. ADME Analysis and Modelling of the Enzyme-Inhibitor Interaction

When the condition of developing a new drug is expected, it is essential to carry out evaluations that can give preliminary information on the compound being studied, with particular regard to absorption, distribution, metabolism and excretion (ADME). For this purpose, we used the web service SwissADME (http://www.swissadme.ch/index.php, accessed on 16 November 2022) [30,31] which was made available by the Swiss Institute of Bioinformatics. 

The evaluation of the interactions between SalB and MMP-9 was performed by using the web service (SwissDock) developed by the Swiss Institute of Bioinformatics in order to predict the most favorable binding modes that may occur between a target protein and a small molecule. This web tool exploits the docking software EADock DSS [32] and the CHARMM force field method for calculation [33]. As a model was used, the crystal structure of MMP-9 complexed with a reverse hydroxamate inhibitor (PDB code: 1GKC), and the docking clusters related to the most favorable interactions with SalB (ZINC entry: 49538628) were visualized and analyzed by using PyMOL Molecular Graphics System.

### 4.3. Fluorometric Inhibition Assay of MMP-9 Activity

The in vitro ability of SalB to influence MMP-9 activity was performed through a fluorometric inhibition assay. For this purpose, we used the recombinant catalytic domain of MMP-9 (CDMMP-9) which represents the 39 kDa active site of the protein (aa 107–457 + NT His Tag). The enzymatic residue was supplied as lyophilized powder which was reconstituted following manufacturer’s instructions with pre-chilled 30% glycerol solution to 10 U/µL. The rapid and sensitive determination of CDMMP-9 proteolytic potential in the presence of increasing concentrations of SalB (from 5 µM to 100 µM) was evaluated with a spectrofluorimetric method, as previously reported [10]. Briefly, the methodology is based on monitoring the ability of the enzyme to specifically cleave the self-quenched synthetic substrate MOCAc-Pro-Leu-Gly-Leu-A2pr(Dnp)-Ala-Arg-NH2 (Peptide Institute INC, Osaka, Japan).

The apparent Michaelis constant (K_M_^app^) values for the interaction between the enzyme and different SalB concentrations were calculated by using the double reciprocal plots of 1/V versus 1/[S]. Such parameters were then used to draw a secondary plot for the determination of the apparent inhibition constant (K_i_). 

### 4.4. Identification of the Experimental Conditions and Cell Culture

The The MDA-MB-231 human breast cancer cells were obtained from the American Type Culture Collection (ATCC) and maintained in exponential growth in DMEM supplemented with 10% heat-inactivated FBS, 100 U/mL penicillin, 100 mg/mL streptomycin and 2 mM glutamine, and kept in a humidified atmosphere with 5% CO_2_ at 37 °C. Cellular viability was determined by trypan blue exclusion assay.

The concentration range and the timing of the cellular treatment with the SalB have been defined through the MTT colorimetric approach which measures viable cells by assessing the conversion of MTT into formazan crystals by mitochondrial activity [34]. Exponentially growing cells were plated in 96-well plates and after 24 h, the medium was replaced with fresh medium and cells were treated with SalB in the range of concentration from 5 µM to 100 µM for 12 h, 24 h, 48 h and 72 h. Negative controls received the same amount of DMSO (in the ratio 1:1000 in the culture medium) used to solubilize the SalB administered in the other wells. At the end of each incubation period, MTT was added to each well at the final concentration of 0.5 mg/mL and incubated at 37 °C for 3 h. The reaction responsible for the formazan release was stopped by the addition of 0.04 N isopropanol, the absorbance was measured at 570 nm in a microplate reader (Biorad, Hercules, CA, USA). 

After defining the parameters for cell treatment with SalB, cells were seeded in six well culture dishes at a density of 5 × 10^4^ cells/well. After 24 h, the medium was replaced with fresh medium and cells were treated with 5 µM (Sal-5) and 50 µM (Sal-50) of SalB. Control cells (CTR) received DMSO in a concentration equal to that used for the solubilization of Sal-5 and Sal-50. After 24 h and 48 h, conditioned media were collected, aliquoted and stored at −20 °C until analysis. 

### 4.5. Evaluation of Gelatinases Activity by Zymography and Determination of TIMPs Expression by Reverse Zymography

Protein concentration in conditioned media was determined by Bradford protein concentration assay. Volumes of each sample corresponding to 10 µg of total proteins were diluted in a non-reducing sample buffer without heating and resolved by 8% SDS-PAGE containing 0.3 mg/mL type B gelatin. The gels were then incubated for 45 min in a renaturation buffer (50 mM Tris-HCl pH 8.0, containing 2.5% Triton X-100) to remove SDS. Subsequently, a 24 h incubation in the developing buffer (50 mM Tris-HCl pH 8.0, containing 5 mM CaCl_2_, 200 mM NaCl and 0.02% Brij 35) was performed to allow enzyme renaturation and activity. Gels were then stained in a 0.1% solution of Coomassie Blue R250 in 40% (*v/v*) methanol and 10% (*v/v*) acetic acid. This analysis was preceded by preliminary evaluations, in which the supernatant of HT1080 fibrosarcoma cells was used as a reference standard for both MMP-2 and MMP-9, as recommended by Toth et al. [35]. Furthermore, in order to verify the metallo-protease nature of the activity displayed, were carried out tests in which the incubation of the zymographic gels was performed in a developing buffer in which a chelating agent (EDTA 100 mM) was added (in this condition no degradation of the substrate was found). Quantitative analysis of visualized spots was performed by using ImageJ software [36]. 

To reveal the presence of TIMPs in collected samples, reverse zymography was performed as previously described with a modification [37]. Briefly, a cell conditioned medium rich in gelatinases able to degrade gelatin during the incubation in the developer buffer was added to the gel. The presence of TIMPs was visualized as dark bands in which the TIMP bound to the enzyme inhibits its gelatinolytic activity. 

### 4.6. Western Blotting Analysis of MMP-9 and TIMP-1

Samples of conditioned media containing 40 µg of total proteins, were mixed with a reducing sample buffer and subjected to 10% SDS-PAGE. Separated proteins were then trans-blotted onto polyvinilidene difluoride (PVDF) transfer membranes. The non-specific protein binding site on membranes was blocked by incubation in a solution containing 5% non-fat dry milk (Biorad, Milan, Italy) in TBS 0.1% Tween 20 (TBS-T) for 1 h, and then incubated overnight at 4 °C with the primary antibodies for human MMP-9 and TIMP-1 diluted in 1% non-fat dry milk in TBS-T. Membranes were then washed in TBS-T, incubated for 1 h with secondary HRP-conjugated antibody diluted in blocking solution, and the immunoreactive bands were detected by inducing a chemiluminescence reaction through the ECL chemiluminescent reagent (GE Healthcare, Little Chalfont, England). Quantitative analysis of immunoreactive spots was performed by using ImageJ software [36].

### 4.7. RNA Purification and Reverse Transcriptase Polymerase Chain Reaction (RT-PCR)

Total RNA was extracted from treated cells with EUROGOLD Total RNA Mini kit (EuroClone, Milan, Italy) and 1 μg of each sample was subjected to retrotranscriptase reaction and PCR amplification in the same tube using the HyperscriptTM One-step RT-PCR Premix (TEMA RICERCA, Bologna, Italy) and following manufacturer’s indications. Expression levels of MMP-9 and TIMP-1 were normalized to the expression levels of the housekeeping β-actin gene. PCR was performed by using the following oligonucleotides: MMP-9 (sense 5′-CGC AGA CAT CGT CAT CCA GT-3′, anti-sense 5′-GGA TTG GCG TTG GAA GAT GA-3′), TIMP-1 (sense 5′-CTG TTG TTG CTG TGG CTG ATA-3′, antisense 5′-CCG TCC ACA AGC AAT GAG T-3′) and β-actin (sense 5′-ATG ATG ATA TCG CCG CGC TCG-3′, antisense 5′-GCG CTC GGT GAG GAT CTT CA-3′). The RT-PCR products were resolved by 1.5% agarose gel electrophoresis. 

### 4.8. Statistical Analysis

Data were statistically analyzed by using SigmaPlot 12.0 software (Systat software, Inc., San Jose, CA, USA) for Windows operating system. Differences between means were evaluated by Student’s t-test with confidence levels set at 95% (*p* < 0.05) and 99% (*p* < 0.01). 

## 5. Conclusions

In the present research, for the first time to our knowledge, the ability of salvianolic acid B, an active compound of *Salviae mitiorrhizae*, to modulate the function of MMP-9 in human MDA-MB-231 breast cancer cells was demonstrated in an in vitro model characterized by a high invasive potential. Particularly, we found a marked tendency of the natural compound to interact with the catalytic domain of MMP-9 with the consequent inhibition of its activity, presumably reducing the ability of the enzyme to interact with the substrate. Furthermore, an inhibitory effect on the enzyme expression was observed. From this point of view, more specific studies will have to be conducted in order to characterize the biochemical mechanisms that oversee this phenomenon.

## Figures and Tables

**Table 1:** SwissADME (absorption, distribution, metabolism and excretion) parameters attributed to SalB.

Properties	Parameters	SalB
Physicochemical Properties	MW	718.61 g/mol
	Rotatable bonds	14
	HBA	16
	HBD	9
	Molar refractivity	178.07
	TPSA	278.04 Å
Lipophilicity (Log *P_o/w_*)	iLOGP	2.10
	XLOGP3	3.98
	WLOGP	2.90
	MLOGP	0.25
	SILICOS-IT	2.57
	Consensus estimation	2.36
Pharmacokinetics	GI absorption	Low
	BBB permeant	No
	P-gp substrate	No
	CYP1A2 inhibitor	No
	CYP2C19 inhibitor	No
	CYP2C9 inhibitor	No
	CYP2D6 inhibitor	No
	CYP3A4 inhibitor	No
	Log *K_p_* (skin permeation)	−7.86 cm/s

MW = molecular weight; HBA = H-bond acceptors; HBD = H-bond donors; TPSA = Topological Polar Surface Area; GI = gastrointestinal; BBB = blood–brain barrier; P-gp = permeability glycoprotein; iLOGP, XLOGP3, WLOGP, MLOGP, SILICOS-IT represent lipophilicity predictive models [Daina et al. 2017]; CYP1A2, CYP2C19, CYP2C9, CYP2D6, CYP3A4 are isoforms of cytochrome P450.

## Data Availability

The datasets used and/or analyzed during the current study are available from the corresponding author on reasonable request.

## References

[1] Nagase H., Visse R., Murphy G. (2006). Structure and function of matrix metalloproteinases and TIMPs. Cardiovasc. Res..

[2] Brew K., Dinakarpandian D., Nagase H. (2000). Tissue inhibitors of metalloproteinases: Evolution, structure and function. Biochim. et Biophys. Acta (BBA) Protein Struct. Mol. Enzym..

[3] Klein T., Bischoff R. (2011). Physiology and pathophysiology of matrix metalloproteases. Amino Acids.

[4] Stamenkovic I. (2000). Matrix metalloproteinases in tumor invasion and metastasis. Semin. Cancer Biol..

[5] Merdad A., Karim S., Schulten H.J., Dallol A., Buhmeida A., Al-Thubaity F., Gari M.A., Chaudhary A.G., Abuzenadah A.M., Al-Qahtani M.H. (2014). Expression of matrix metalloproteinases (MMPs) in primary human breast cancer: MMP-9 as a potential biomarker for cancer invasion and metastasis. Anticancer. Res..

[6] Giusti I., D’Ascenzo S., Millimaggi D., Taraboletti G., Carta G., Franceschini N., Pavan A., Dolo V. (2008). Cathepsin B mediates the pH-dependent proinvasive activity of tumor-shed microvesicles. Neoplasia.

[7] Nissinen L., Kähäri V.M. (2014). Matrix metalloproteinases in inflammation. Biochim. Biophys. Acta, Gen. Subj..

[8] Lee M., Celenza G., Boggess B., Blasé J., Shi Q., Toth M., Margarida Bernardo M., Wolter W.R., Suckow M.A., Hesek D. (2009). A Potent Gelatinase Inhibitor with Anti-Tumor-Invasive Activity and its Metabolic Disposition. Chem. Biol. Drug Des..

[9] Jacobsen J.A., Jourden J.L.M., Miller M.T., Cohen S.M. (2010). To bind zinc or not to bind zinc: An examination of innovative approaches to improved metalloproteinase inhibition. Biochim. Biophys. Acta, Mol. Cell Res..

[10] Ianni A., Celenza G., Franceschini N. (2019). Oxaprozin: A new hope in the modulation of matrix metalloproteinase 9 activity. Chem. Biol. Drug Des..

[11] Huneif M.A., Alqahtani S.M., Abdulwahab A., Almedhesh S.A., Mahnashi M.H., Riaz M., Ur-Rahman N., Jan M.S., Ullah F., Aasim M. (2022). α-Glucosidase, α-Amylase and Antioxidant Evaluations of Isolated Bioactives from Wild Strawberry. Molecules.

[12] Al-Joufi F.A., Jan M., Zahoor M., Nazir N., Naz S., Talha M., Sadiq A., Nawaz A., Khan F.A. (2022). Anabasis articulata (Forssk.) Moq: A Good Source of Phytochemicals with Antibacterial, Antioxidant, and Antidiabetic Potential. Molecules.

[13] Majid M., Farhan A., Asad M.I., Khan M.R., Hassan S.S.U., Haq I.U., Bungau S. (2022). An Extensive Pharmacological Evaluation of New Anti-Cancer Triterpenoid (Nummularic Acid) from Ipomoea batatas through In Vitro, In Silico, and In Vivo Studies. Molecules.

[14] Ende C., Gebhardt R. (2004). Inhibition of matrix metalloproteinase-2 and-9 activities by selected flavonoids. Planta Med..

[15] Rice-Evans C. (2004). Flavonoids and isoflavones: Absorption, metabolism, and bioactivity. Free Radic. Biol. Med..

[16] Wang B.Q. (2010). Salvia miltiorrhiza: Chemical and pharmacological review of a medicinal plant. J. Med. Plants Res..

[17] Zhao G.R., Zhang H.M., Ye T.X., Xiang Z.J., Yuan Y.J., Guo Z.X., Zhao L.B. (2008). Characterization of the radical scavenging and antioxidant activities of danshensu and salvianolic acid B. Food Chem. Toxicol..

[18] Zhang H.S., Wang S.Q. (2006). Salvianolic acid B from Salvia miltiorrhiza inhibits tumor necrosis factor-α (TNF-α)-induced MMP-2 upregulation in human aortic smooth muscle cells via suppression of NAD(P)H oxidase-derived reactive oxygen species. J. Mol. Cell. Cardiol..

[19] Jiang B., Chen J., Xu L., Gao Z., Deng Y., Wang Y., Xu F., Shen X., Guo D.A. (2010). Salvianolic acid B functioned as a competitive inhibitor of matrix metalloproteinase-9 and efficiently prevented cardiac remodeling. BMC Pharmacol..

[20] Wang Y., Xu F., Chen J., Shen X., Deng Y., Xu L., Yin J., Chen H., Teng F., Liu X. (2011). Matrix metalloproteinase-9 induces cardiac fibroblast migration, collagen and cytokine secretion: Inhibition by salvianolic acid B from Salvia miltiorrhiza. Phytomedicine.

[21] Sha W., Zhou Y., Ling Z.Q., Xie G., Pang X., Wang P., Gu X. (2018). Antitumor properties of Salvianolic acid B against triple-negative and hormone receptor-positive breast cancer cells via ceramide-mediated apoptosis. Oncotarget.

[22] Katary M.A., Abdelsayed R., Alhashim A., Abdelhasib M., Elmarakby A.A. (2019). Salvianolic acid B slows the progression of breast cancer cell growth via enhancement of apoptosis and reduction of oxidative stress, inflammation, and angiogenesis. Int. J. Mol. Sci..

[23] Varma M.V., Sateesh K., Panchagnula R. (2005). Functional role of P-glycoprotein in limiting intestinal absorption of drugs: Contribution of passive permeability to P-glycoprotein mediated efflux transport. Mol. Pharm..

[24] Testa B., Kraemer S.D. (2007). The Biochemistry of Drug Metabolism—An Introduction. Chem. Biodivers.

[25] Kirchmair J., Göller A.H., Lang D., Kunze J., Testa B., Wilson I.D., Glen R.C., Schneider G. (2015). Predicting drug metabolism: Experiment and/or computation?. Nat. Rev. Drug. Discov..

[26] Liu Q., Loo W.T., Sze S.C.W., Tong Y. (2009). Curcumin inhibits cell proliferation of MDA-MB-231 and BT-483 breast cancer cells mediated by down-regulation of NFκB, cyclinD and MMP-1 transcription. Phytomedicine.

[27] Lin S.J., Lee I.T., Chen Y.H., Lin F.Y., Sheu L.M., Ku H.H., Shiao M.S., Chen J.W., Chen Y.L. (2007). Salvianolic acid B attenuates MMP-2 and MMP-9 expression in vivo in apolipoprotein-E-deficient mouse aorta and in vitro in LPS-treated human aortic smooth muscle cells. J. Cell. Biochem..

[28] Dai J.P., Zhu D.X., Sheng J.T., Chen X.X., Li W.Z., Wang G.F., Li K.S., Su Y. (2015). Inhibition of tanshinone IIA, salvianolic acid A and salvianolic acid B on areca nut extract-induced oral submucous fibrosis in vitro. Molecules.

[29] Cayetano-Salazar L., Nava-Tapia D.A., Astudillo-Justo K.D., Arizmendi-Izazaga A., Sotelo-Leyva C., Herrera-Martinez M., Villegas-Comonfort S., Navarro-Tito N. (2022). Flavonoids as regulators of TIMPs expression in cancer: Consequences, opportunities, and challenges. Life Sci..

[30] Hassan S.S.U., Abbas S.Q., Ali F., Ishaq M., Bano I., Hassan M., Jin H.Z., Bungau S.G. (2022). A Comprehensive in silico exploration of pharmacological properties, bioactivities, molecular docking, and anticancer potential of vieloplain F from Xylopia vielana Targeting B-Raf Kinase. Molecules.

[31] Daina A., Michielin O., Zoete V. (2017). SwissADME: A free web tool to evaluate pharmacokinetics, drug-likeness and medicinal chemistry friendliness of small molecules. Sci. Rep..

[32] Grosdidier A., Zoete V., Michielin O. (2011). SwissDock, a protein-small molecule docking web service based on EADock DSS. Nucleic Acids Res..

[33] Vanommeslaeghe K., Hatcher E., Acharya C., Kundu S., Zhong S. (2010). CHARMM general force field: A force field for drug-like molecules compatible with the CHARMM all-atom additive biological force fields. J. Comput. Chem..

[34] Mosmann T. (1983). Rapid colorimetric assay for cellular growth and survival: Application to proliferation and cytotoxicity assays. J. Immunol. Met..

[35] Toth M., Sohail A., Fridman R. (2012). Assessment of gelatinases (MMP-2 and MMP-9) by gelatin zymography. Metastasis Research Protocols.

[36] Rasband W.S. (2012). ImageJ software.

[37] Hawkes S.P., Li H., Taniguchi G.T. (2010). Zymography and reverse zymography for detecting MMPs and TIMPs. Matrix Metalloproteinase Protocols.

